# Combining anatomical and biochemical markers in the detection and risk stratification of coronary artery disease

**DOI:** 10.1093/ehjci/jeae093

**Published:** 2024-04-09

**Authors:** Miriam Albus, Tobias Zimmermann, Daniela Median, Klara Rumora, Ganna Isayeva, Melissa Amrein, Ibrahim Schaefer, Joan Walter, Evita Michel, Gabrielle Huré, Ivo Strebel, Federico Caobelli, Philip Haaf, Simon M Frey, Christian Mueller, Michael J Zellweger

**Affiliations:** Cardiovascular Research Institute Basel (CRIB) and Department of Cardiology, University Hospital Basel, University of Basel, Petersgraben 4, CH-4031, Basel, Switzerland; Department of Internal Medicine, University Hospital Basel, University of Basel, Basel, Switzerland; Cardiovascular Research Institute Basel (CRIB) and Department of Cardiology, University Hospital Basel, University of Basel, Petersgraben 4, CH-4031, Basel, Switzerland; Departement of Anesthesiology and Intensive Care, University Hospital Basel, University of Basel, Basel, Switzerland; Cardiovascular Research Institute Basel (CRIB) and Department of Cardiology, University Hospital Basel, University of Basel, Petersgraben 4, CH-4031, Basel, Switzerland; Cardiovascular Research Institute Basel (CRIB) and Department of Cardiology, University Hospital Basel, University of Basel, Petersgraben 4, CH-4031, Basel, Switzerland; Cardiovascular Research Institute Basel (CRIB) and Department of Cardiology, University Hospital Basel, University of Basel, Petersgraben 4, CH-4031, Basel, Switzerland; Cardiovascular Research Institute Basel (CRIB) and Department of Cardiology, University Hospital Basel, University of Basel, Petersgraben 4, CH-4031, Basel, Switzerland; Cardiovascular Research Institute Basel (CRIB) and Department of Cardiology, University Hospital Basel, University of Basel, Petersgraben 4, CH-4031, Basel, Switzerland; Department of Internal Medicine, University Hospital Basel, University of Basel, Basel, Switzerland; Cardiovascular Research Institute Basel (CRIB) and Department of Cardiology, University Hospital Basel, University of Basel, Petersgraben 4, CH-4031, Basel, Switzerland; Department of Medical Oncology and Hematology, University Hospital Zurich, University of Zurich, Zürich, Switzerland; Cardiovascular Research Institute Basel (CRIB) and Department of Cardiology, University Hospital Basel, University of Basel, Petersgraben 4, CH-4031, Basel, Switzerland; Cardiovascular Research Institute Basel (CRIB) and Department of Cardiology, University Hospital Basel, University of Basel, Petersgraben 4, CH-4031, Basel, Switzerland; Cardiovascular Research Institute Basel (CRIB) and Department of Cardiology, University Hospital Basel, University of Basel, Petersgraben 4, CH-4031, Basel, Switzerland; Department of Radiology and Nuclear Medicine, University Hospital Basel, University of Basel, Basel, Switzerland; Cardiovascular Research Institute Basel (CRIB) and Department of Cardiology, University Hospital Basel, University of Basel, Petersgraben 4, CH-4031, Basel, Switzerland; Cardiovascular Research Institute Basel (CRIB) and Department of Cardiology, University Hospital Basel, University of Basel, Petersgraben 4, CH-4031, Basel, Switzerland; Cardiovascular Research Institute Basel (CRIB) and Department of Cardiology, University Hospital Basel, University of Basel, Petersgraben 4, CH-4031, Basel, Switzerland; Cardiovascular Research Institute Basel (CRIB) and Department of Cardiology, University Hospital Basel, University of Basel, Petersgraben 4, CH-4031, Basel, Switzerland

**Keywords:** coronary artery disease, Agatston score, calcium score, myocardial perfusion SPECT, myocardial ischaemia, high-sensitivity cardiac troponin, prognosis

## Abstract

**Aims:**

We aimed to test the hypothesis if combining coronary artery calcium score (Ca-score) as a quantitative anatomical marker of coronary atherosclerosis with high-sensitivity cardiac troponin as a quantitative biochemical marker of myocardial injury provided incremental value in the detection of functionally relevant coronary artery disease (fCAD) and risk stratification.

**Methods and results:**

Consecutive patients undergoing myocardial perfusion single-photon emission computed tomography (MPS) without prior CAD were enrolled. The diagnosis of fCAD was based on the presence of ischaemia on MPS and coronary angiography; fCAD was centrally adjudicated in the diagnostic and prognostic domain. Diagnostic accuracy was evaluated using the area under the receiver-operating characteristic curve (AUC). The composite of cardiovascular death and non-fatal acute myocardial infarction (AMI) within 730 days was the primary prognostic endpoint. Among 1715 patients eligible for the diagnostic analysis, 399 patients had fCAD. The combination of Ca-score and high-sensitivity cardiac troponin T (hs-cTnT) had good diagnostic accuracy for the diagnosis of fCAD (AUC 0.79, 95% confidence interval (CI) 0.77–0.81), but no incremental value compared with the Ca-score alone (AUC 0.79, 95% CI 0.77–0.81, *P* = 0.965). Similar results were observed using high-sensitivity cardiac troponin I (AUC 0.80, 95% CI 0.77–0.82) instead of hs-cTnT. Among 1709 patients (99.7%) with available follow-up, 59 patients (3.5%) suffered the composite primary prognostic endpoint (non-fatal AMI, *n* = 34; CV death, *n* = 28). Both Ca-score and hs-cTnT had independent prognostic value. Increased risk was restricted to patients with elevation in both markers.

**Conclusion:**

The combination of the Ca-score with hs-cTnT increases the prognostic accuracy for future events but does not provide incremental value vs. the Ca-score alone for the diagnosis of fCAD.

**Study registration:**

Clinical trial registration: NCT00470587.

## Graphical Abstract

**Graphical Abstract:**
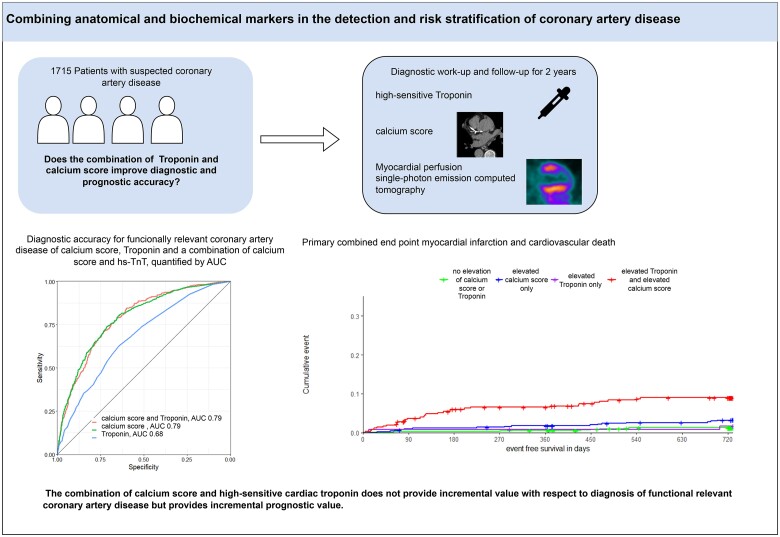

## Introduction

Multiple cardiac imaging modalities are used to evaluate patients in the coronary artery disease (CAD) setting.^1–3^ Due to cardiac imaging examinations, annual costs of more than 500 million dollars accumulate in the USA alone. Up to 80% of these tests turn out to be normal, an increasing trend that has been observed over the last years.^4^ Associated with this trend are increasing healthcare costs and exposure of the patient to radiation and/or contrast agents.^5,6^

Simple, safe, and cost-efficient diagnostic pathways for patients with suspected functionally relevant CAD (fCAD) still remain an unmet clinical need.

The coronary artery calcium score (Ca-score) performed by non-contrast computed tomography (CT) provides a quantitative assessment of coronary artery calcification (Agatston score).^7^ The radiation dose to asses a Ca-score is low (0.2–0.4 mSv)^8^ and its costs are relatively low.^7,9^ Previous studies have shown that the Ca-score has high diagnostic and prognostic value.^10–12^ Nevertheless, usually, the Ca-score is used in combination with further anatomical or functional testing and generally is not recommended as a stand-alone method.^1^

Therefore, it seems attractive to combine the Ca-score with biochemical markers to still improve its diagnostic and prognostic accuracy. To the best of our knowledge, such an approach has not yet been evaluated.

We, therefore, hypothesized that combining the Ca-score with either high-sensitivity cardiac troponin T (hs-cTnT) or high-sensitivity cardiac troponin I (hs-cTnI) as quantitative markers of myocardial injury might provide incremental diagnostic and prognostic value. As myocardial ischaemia is one of the triggers of myocardial release of hs-cTnT and hs-cTnI, its combination with the Ca-score might be helpful for the cost-effective detection of fCAD.^13–15^

Hs-cTnT and hs-cTnI are the most important biomarkers used in the acute CAD setting [acute myocardial infarction (AMI)]^16^ but are still not an established part of the routine clinical diagnostics in the setting of the chronic coronary syndrome.^1^ However, pilot studies have indicated possible diagnostic and prognostic value of hs-cTnT and hs-cTnI for CAD and major cardiac adverse events (MACE).^13–15,17,18^

The aim of our study therefore was to evaluate the diagnostic and prognostic value of a combined approach utilizing the Ca-score and hs-cTnT or hs-cTnI.

## Methods

### Study design and overview

This analysis is part of a large diagnostic study in which data were collected prospectively (NCT01838148, clinicaltrials.gov), designed to promote the early detection of fCAD.^13,15,19^ The study was approved by the local ethics committee and carried out according to the principles of the Declaration of Helsinki. All patients provided written informed consent. The authors designed this study, gathered, analysed, and reported the data according to the STARD guidelines.^20^

### Patient population

From April 2011 to May 2016, consecutive patients without known CAD but suspected fCAD referred for rest/stress myocardial perfusion single-photon emission computed tomography (MPS) to the University Hospital Basel, Switzerland were recruited. This study was performed in an all-comers population without specific exclusion criteria. For this analysis, however, we excluded patients with terminal kidney failure requiring dialysis and patients without available Ca-score or hs-cTnT measurement.

### Measurement of the Ca-score

Coronary artery calcium was measured using non-contrast CT. The measurement of coronary artery calcium was routinely performed for patients without known coronary artery disease using the Symbia-Siemens® multi-detector-CT system and the Ca-score was calculated as described by Agatston.^7^ Images were processed with the Syngo MI Applications® software.

### Clinical pre-/post-test probability of fCAD

For each patient, the treating cardiologist recorded a clinical assessment regarding the presence of fCAD on a visual analogue scale (VAS) with values between 0% and 100%. Clinical pre-test probability was obtained before stress testing and was based on all medical information available at this point of time (age, sex, history of cardiovascular disease, cardiovascular risk factors, symptoms, physical examination, and baseline electrocardiogram (ECG) data). After stress testing, a second clinical assessment was recorded (clinical post-test probability), including symptoms during exercise/stress, achieved workload, and potential ECG changes recorded during exercise/stress. The cardiologist was blinded to the results of biomarker measurements and MPS images throughout the assessment.

### Blood sampling and laboratory methods

For all patients, venous blood samples were collected at the time of presentation for MPS. The measurements of hs-cTnT and hs-cTnI were performed in the core laboratory of our institution.

Samples underwent centrifugation and were either measured directly from fresh samples or frozen at −80°C until measurement.

Hs-cTnT was analysed with the Elecsys assay platform (Roche). The upper limit of normal (99th percentile) for this assay is 14 ng/L.^21^ Hs-cTnI was measured with the Architect assay platform (Abbott), with an upper limit of normal of 26.2 ng/L.^22^

Blood samples were processed by laboratory technicians blinded to any patient data.

### Adjudication of the presence of fCAD

During the recruitment period of this study, MPS was the preferred imaging method at our institution for patients with a wide range of pre-test probability of suspected fCAD. Adjudication of fCAD was based on expert interpretation of MPS images. However, results of MPS interpretation were potentially overruled by information obtained from invasive coronary angiography and fractional flow reserve measurements whenever available and divergent, as those methods are currently considered the gold standard for diagnosis of CAD.

All patients underwent a routine rest/stress dual isotope (201Tl for rest, 99mTc sestamibi for stress) or single isotope (99mTc sestamibi for stress and rest) MPS protocol as described previously.^19,23^ In short, we performed a routine stepwise exercise protocol or pharmacologic stress with continuous electrocardiographic monitoring. MPS images were scored semi-quantitatively using a 17-segment model with a 5-point scale. Summed stress score (SSS) and summed rest score (SRS) were calculated by adding the scores of the 17 segments in the stress and rest images. SSS and SRS were evaluated by two independent readers using visual assessment and compared with the software (QGS) result. Summed difference score (SDS) represents the difference between SSS and SRS scores. FCAD was defined as a SDS of at least 2 (3% of myocardium) or a positive transient ischaemic dilation ratio. In case of equivocal findings from MPS and coronary angiography, two independent cardiologists (one interventional cardiologist and one general cardiologist) reviewed the case. The result of the MPS was overruled by coronary angiographic findings, if coronary angiography within 3 months showed divergent results. A positive coronary angiography was defined as follows: (i) a high-grade coronary lesion (>75%) or (ii) a 50–75% stenosis followed by a percutaneous coronary intervention or a coronary artery bypass or if there was fractional flow reserve lower than 0.80 (except in the case of AMI).

MPS images and coronary angiography findings were evaluated blinded to all biomarker concentrations.

### Follow-up and prognostic outcome definition

Follow-up was performed by letter or standardized telephone interview after 1 and 2 years. If the patient reported cardiovascular events, further information was obtained from hospital records, general practitioner/cardiologist records, or the national death registry. All deaths not clearly associated with non-cardiovascular reasons were defined as cardiovascular deaths.

This analysis was conducted with the 2-year follow-up data. The primary endpoint for the prognostic analysis was a combined outcome measure of cardiovascular death and AMI. The secondary endpoints were (i) all-cause death; (ii) a combined outcome measure of cardiovascular death, AMI, and late revascularization; and (iii) a combined outcome measure of peripheral arterial event and stroke or transient ischaemic attack (TIA). Late revascularization excluded revascularization within 60 days after the primary presentation for MPS.^24,25^

### Statistical analysis

Continuous variables are presented as median and interquartile range. Categorical variables are presented as numbers and percentages. The normality assumption was tested by the Kolmogorov–Smirnov test and visual *Q*–*Q* plot analysis. Inter-group differences of the baseline characteristics were compared using the Fisher exact test for categorical variables and the Mann–Whitney *U* test for continuous variables.

Univariate and bivariate logistic regression analysis was performed using log2-transformed values of hs-cTn and the Ca-score. Area under the receiver-operating characteristic curve (AUC) was calculated to quantify the diagnostic accuracy of hs-cTnT, Ca-score, and a combination of them for an adjudicated diagnosis of fCAD. Comparison of AUCs was performed according to the method described by DeLong *et al*.^26^ A sub-analysis was performed for a subset of patients with hs-cTnI measurement and for the subset of patients with available clinical pre-/post-test probability of fCAD. Additionally, a subgroup analysis was performed considering men and women separately to find sex-specific differences in the performance.

The influence of hs-cTnT and the Ca-score to predict the pre-defined cardiovascular outcomes was calculated using Cox regression analysis: in a first model using only hs-cTnT and the Ca-score and in a second model, to adjust for different baseline characteristics in the two groups, additionally including age, sex, renal function (Cystatin C), and history of cardiovascular diseases [atrial fibrillation, heart failure, peripheral arterial occlusive disease, stroke/TIA, and pacemaker]. Confounders were chosen due to clinical plausibility and known influence.^27,28^ For adjusted models, patients with missing values of Cystatin C were excluded.

Kaplan–Meyer curves were generated using the combination of Ca-score and hs-cTnT, as binary variables respectively below and above a pre-defined and clinically relevant cut-off. As a clinically relevant cut-off, a hs-cTnT value above the upper limit of normal (14 ng/L, 99th percentile) was used. For the Ca-score, the analysis was performed for two different clinically relevant cut-offs: for the main analysis, we used a cut-off above 100 (approximate median value of our cohort),^29–34^ and for a sub-analysis, we used a cut-off value of 0.^12,33^ Cox regression analysis was performed for the groups of patients classified by a binary measure of hs-cTnT and Ca-score with adjustment accordingly to the model using hs-cTnT and Ca-score as continuous parameters (see above).

All hypothesis testing was two-tailed, and a *P*-value of <0.05 was considered statistically significant.

Statistical analyses were performed with R version 4.0.3.

## Results

### Patient characteristics

A total of 2242 consecutive patients without known coronary artery disease were enrolled in our study. Of these patients, 1715 patients were eligible for our primary diagnostic analysis (see Supplementary data online, *Figure S1*). For the prognostic analysis, we excluded patients with missing follow-up data (6/1715 patients, 0.3%).

Of the 1715 eligible patients for the primary analysis, 400 (23.3%) patients underwent coronary angiography within 3 months, with 5/400 patients (0.3%) being reclassified to the fCAD group and 56/400 patients (3.3%) being reclassified to the non-fCAD group.

The baseline characteristics of patients assigned to the group of existent fCAD were different to those of patients without fCAD. They were more likely to be male and older and had more comorbidities. The percentage of symptomatic patients was similar in both groups (*Table 1*).

**Table 1:** Baseline characteristics according to presence (+) or absence (−) of fCAD

	Overall	fCAD−	fCAD+
	*n* = 1715	*n* = 1316	*n* = 399
Sex, female	753 (43.9%)	651 (49.5%)	102 (25.6%)
Age	68 (59–76)	67 (58–74)	72 (65–78)
BMI	26.9 (24.2–30.5)	26.8 (24.1–30.5)	27.4 (24.7–30.5)
Symptoms	1019 (59.4%)	782 (59.4%)	237 (59.4%)
Pre-existing disease			
Arterial hypertension	1219 (71.1%)	895 (68%)	324 (81.2%)
Hypercholesterolaemia	906 (52.8%)	662 (50.3%)	244 (61.2%)
Arterial fibrillation	248 (14.5%)	176 (13.4%)	72 (18%)
Heart failure	26 (1.5%)	16 (1.2%)	10 (2.5%)
Diabetes	346 (20.2%)	240 (18.2%)	106 (26.6%)
Peripheral arterial disease	101 (5.9%)	64 (4.9%)	37 (9.3%)
Status post-stroke/TIA	130 (7.6%)	77(5.9%)	53 (13.3%)
Pacemaker/ICD/CRT	41 (2.4%)	28 (2.13%)	13 (3.3%)
Family history	390 (22.7%)	305 (23.2%)	85 (21.3%)
Malignancy	255 (14.9%)	196 (14.9%)	59 (14.8%)
Nicotine use	919 (53.6%)	679 (51.6%)	240 (60.1%)
Stopped use	588 (34.3%)	420 (31.9%)	168 (42.1%)
Active use	331 (19.3%)	259 (19.7%)	72 (18%)
Beta-blocker	585 (34.1%)	425 (32.3%)	160 (40.1%)
ACE blocker	359 (20.9%)	262 (19.9%)	97 (24.3%)
AT2 antagonists	528 (30.8%)	385 (29.3%)	143 (35.8%)
Diuretics	591 (34.5%)	419 (31.8%)	172 (43.1%)
Anticoagulation	235 (13.7%)	164 (12.5%)	71 (17.8%)
Warfarin	157 (9.2%)	105 (8%)	52 (13%)
New oral anticoagulants	78 (4.5%)	59 (4.5%)	19 (4.8%)
Aspirin	1046 (61%)	478 (36.3%)	191 (47.9%)
Statin	604 (35.2%)	427 (32.4%)	177 (44.4%)
Cystatin C (RFU), *n* = 1654	2418.8 (2076.7–2846.1)	2358.6 (2045.9–2764.3)	2595.8 (2241.7–3186.9)
Stress test modality ergometrie	1115 (65%)	894 (68%)	221 (55.4%)
Rate pressure product at rest ^a^	11115 (9628–12879)	11050 (9549.8–12816)	11502 (9902–13156)
Rate pressure product at stress^a^	30225 (26666–33600)	30432 (26996.8–33852)	29141 (25453–32240)

Continous variables are presented as median [interquartile range (IQR)]; categorical variables are presented as total number (percentage).

^a^For those who had an ergometry stress.

### Diagnostic accuracy for the presence of fCAD

**Figure 1:**
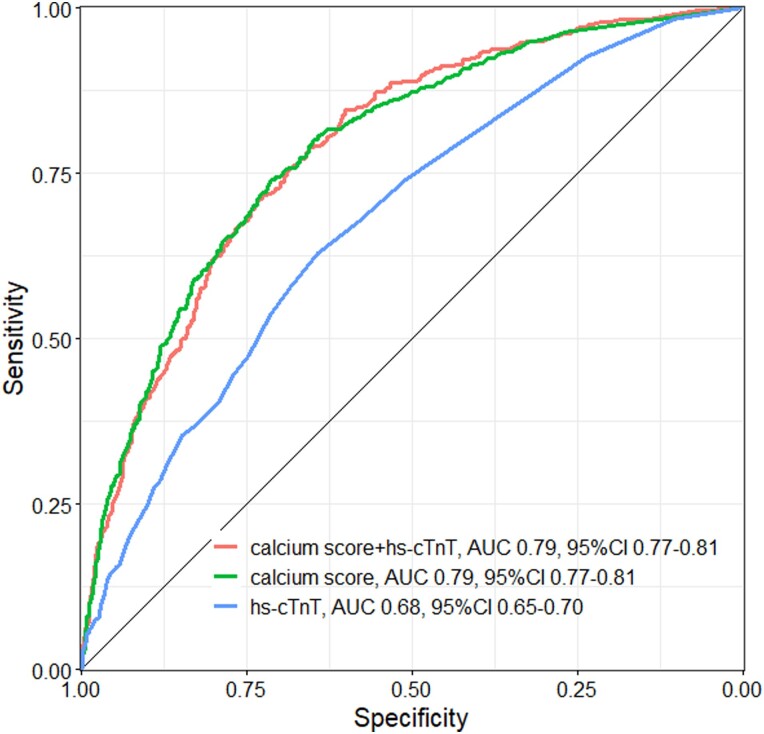
Diagnostic accuracy of calcium score, hs-TnT, and a combination of calcium score and hs-TnT, quantified by AUC.

In our analysis, hs-cTnT alone showed an AUC of 0.68 (95% confidence interval (CI) 0.65–0.70), and the Ca-score alone showed an AUC of 0.79 (95% CI 0.77–0.81) for the presence of fCAD. Performance of the combination of Ca-score and hs-cTnT (0.79, 95% CI 0.77–0.81) was comparable with the AUC of the Ca-score alone (*P* = 0.965) but significantly better than hs-cTnT alone (*P* < 0.001) (*Figure 1*).

### Sex differences

There was a significantly higher median concentration of hs-cTnT/I and a higher Ca-score in male compared with female patients (*Table 2*).

**Table 2:** Baseline values of hs-TnT/I, calcium score, and clinical pretest probability according to sex and presence of fCAD

	Overall			Women			Men			*P*-value*
	Overall *n* = 1715	fCAD+ 23.3%	fCAD− 76.7%	Overall *n* = 753	fCAD+ 13.6%	fCAD− 86.4%	Overall *n* = 962	fCAD+ 30.9%	fCAD− 69.1%	
Calcium score	102 (4–477)	585 (197–1379)	46 (0–271)	34 (0–261)	475 (111–1027)	22 (0–164)	194 (22–675)	623 (227–1527)	101 (7–397)	<0.001
hs-cTnT (ng/L)	8 (5–15)	12 (7–21)	7 (5–13)	7 (4–12)	10 (7–17.8)	7 (4–11)	9 (6–17)	13 (8–23)	8 (5–14)	<0.001
hs-cTnI (ng/L)^a^	3.4 (2–6.2)	5.3 (2–5.3)	3 (2–5.3)	3 (1.9–5)	4.5 (3–8.1)	2.9 (1.7–4.9)	4 (2.1–7.5)	6 (3.3–10.8)	3.2 (2–6)	<0.001
CPTP (%)^a^	30 (20–50)	50 (30–70)	30 (20–50)	30 (20–50)	40 (20–60)	30 (20–50)	30 (20–60)	50 (30–70)	30 (20–50)	0.002

Variables are presented as median (IQR). CPTP, clinical pretest probability.

^a^Distinct *n* (patients with missing value are excluded for this analysis: hs-TnI, *n* = 1636; CPTP, *n* = 1658).

^*^ *P*-value applies to difference between women and men.

A sex-specific approach did not alter the diagnostic performance, and there was no difference in AUC for men (0.76, 95% CI 0.73–0.80) compared with women (0.79, 95% CI 0.74–0.83, *P* = 0.381), neither compared with the main analysis including both sexes (see Supplementary data online, *Figures S2 and S3*).

### Sub-analysis hs-cTnI and clinical pre-/post-test probability

In a sub-analysis in patients with available hs-cTnI measurements, the combination of hs-cTnI and the Ca-score showed similar results (AUC 0.80, 95% CI 0.77–0.82) as the combination of hs-cTnT and the Ca-score (*P* = 0.208) (see Supplementary data online, *Figure S4*). Analysis in patients with available clinical pre-/post-test probability of fCAD showed a significantly better performance of the combination of hs-cTnT and the Ca-score (or the Ca-score alone, but not hs-cTnT alone) compared with the clinical assessment before (AUC 0.64, 95% CI 0.61–0.67, *P* < 0.001) or after stress testing (AUC 0.65, 95% CI 0.61–0.68, *P* < 0.001; Supplementary data online, *Figure S5*).

### Prognostic performance

Follow-up data were available for 1709 patients (99.6%), with a complete 2-year follow-up in 1688 patients (98.4%). During this follow-up period, 59 patients experienced a MACE (AMI, *n* = 34; cardiovascular death, *n* = 28), and 63 patients died (cardiovascular and non-cardiovascular death).

Unadjusted Cox regression analysis (using hs-TnT and Ca-score as a continuous variable) showed significant predictive value for the Ca-score and hs-cTnT regarding all endpoints. For the primary endpoint, we observed a hazard ratio (HR) of 1.11 (95% CI 1.03–1.19, *P* = 0.005) for the Ca-score and 1.54 (95% CI 1.29–1.84, *P* < 0.001) for hs-cTnT per two-fold increase of the respective parameter. For the first secondary endpoint (all-cause death), a HR of 1.15 (95% CI 1.06–1.25, *P* = 0.001) was found for the Ca-score and a HR of 1.68 (95% CI 1.43–1.96, *P* < 0.001) for hs-cTnT (HR for other secondary endpoint: see Supplementary data online, *Table S1A*).

After adjusting for age, sex, renal function, and history of cardiovascular disease, the influence of the Ca-score remained significant for the primary endpoint (HR 1.08, 95% CI 1.00–1.17, *P* = 0.044) and for all of the secondary endpoints, except the combined endpoint of peripheral arterial event and stroke/TIA. Hs-cTnT alone was not independently predictive for the primary endpoint, but overall death (HR 1.33, 95% CI 1.04–1.70, *P* = 0.024) and the combined endpoint of peripheral arterial event and stroke/TIA (see Supplementary data online, *Table S1B*).

**Figure 2:**
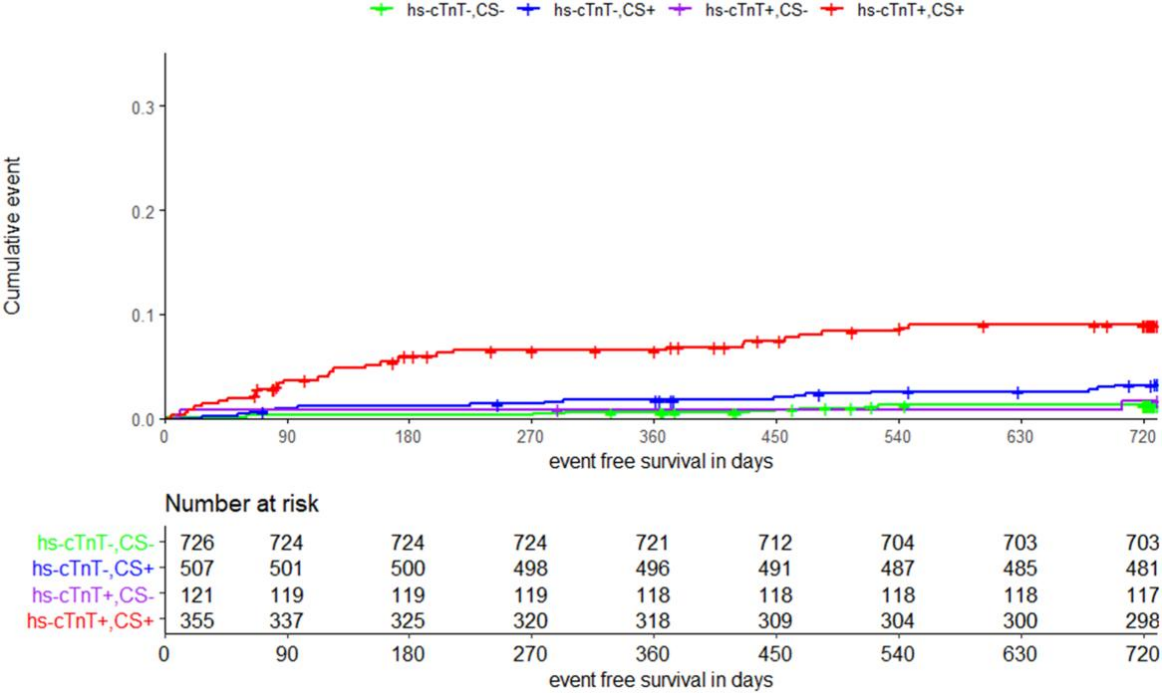
Kaplan–Meier curve for primary endpoint (cardiovascular death, AMI). Groups classified by calcium score (CS) and hs-cTnT below (−) and above (+) the cut of value (cut of value CS < 100; cut of value hs-cTnT < 14 ng/L).

**Figure 3:**
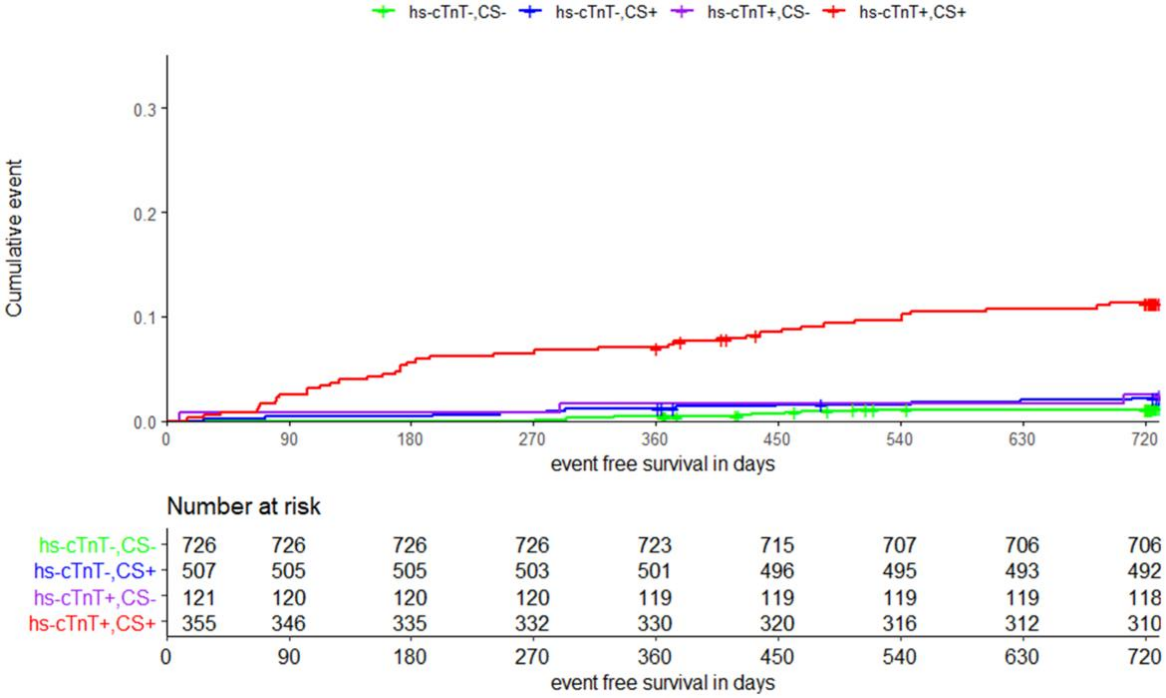
Kaplan–Meier curve for secondary endpoint 1 (all-cause death). Groups classified by calcium score (CS) and hs-cTnT below (−) and above (+) the cut of value (cut of value CS < 100; cut of value hs-cTnT < 14 ng/L).

The four patient groups used in the Kaplan–Meier curves, which were stratified by Ca-score and hs-cTnT, were different in their baseline characteristics (see Supplementary data online, *Table S2*). For the primary endpoint, we observed significantly higher event rates for patients with Ca-score and hs-cTnT above the defined cut-off values (*n* = 31, 8.7%) compared with all other groups. There was no significant difference between patients with just one parameter above the cut-off value (*n* = 17, 3.4%; *n* = 2, 1.6%) compared with patients with both parameters below the cut-off value (*n* = 9, 1.2%) (*Figure 2*). Similar results were observed for the endpoint of all-cause death and the combined endpoint of AMI, cardiovascular death, and late revascularization, with the exception of the latter showing additionally significantly lower event rates for patients with both values below the cut-off (*Figure 3*; Supplementary data online, *Figure S6*). The Kaplan–Meier curves for the combined endpoint of stroke/TIA and peripheral arterial event showed different results with higher event rates in patients of both groups with elevated hs-cTnT (*n* = 7, 5.8% and *n* = 29, 8.1% vs. *n* = 12, 1.7% and *n* = 17, 3.3%) independent of the Ca-score value (see Supplementary data online, *Figure S7*). The group differences remained significant in Cox regression analysis after adjusting for age, sex, renal function, and history of cardiovascular disease (see Supplementary data online, *Table S3*).

A sub-analysis with 0 as the cut-off for the Ca-score showed very distinct numbers of patients for each group. Only 28 patients had a Ca-score of 0 and an elevated hs-cTnT (above the pre-defined value of 14 ng/L). The prognostic trend was similar with regard to the primary and secondary endpoints in the sub-analysis, but some statistically significant differences we observed in the main analysis showed *P*-values above 0.05 in the sub-analysis (see Supplementary data online, *Figures S8–S11*). We observed one event in the group of patients with a Ca-score of 0 and an elevated hs-cTnT (death due to heart failure).

## Discussion

The main findings of our study are as follows: First, the Ca-score provided a good and hs-cTnT/I a moderate diagnostic accuracy for fCAD. Second, a combined diagnostic approach of the Ca-score and hs-cTnT/TnI did not provide any additional diagnostic accuracy compared with the Ca-score alone. Third, the clinical probability before and after the stress testing was less accurate in the diagnostic evaluation than the Ca-score alone or the combined model with hs-TnT. Fourth, the diagnostic performance was similar in male and female patients. Fifth, regarding the primary combined prognostic endpoint of AMI and cardiovascular death, we found a substantially higher event rate for patients with higher Ca-score and elevated hs-cTnT compared with patients with elevation of only one of these variables. Sixth, for the two secondary endpoints of all-cause death and the combined endpoint of AMI, cardiovascular death, and late revascularization, an elevated risk for patients with elevation of both parameters was observed. Seventh, the secondary endpoint of peripheral vascular events (peripheral arterial event and stroke/TIA), in contrast to the cardiac endpoints, showed higher event rates for patients with elevated hs-cTnT concentrations independent of the amount of coronary artery calcium. Eight, the prognostic sub-analysis using a cut-off value of 0 for the Ca-score showed that only a very small number of patients with a Ca-score of 0 had an elevated hs-cTnT level.

Our cohort represents a real-world setting of patients referred for further imaging workup for suspected fCAD.

The diagnostic performance of hs-cTnT/I and of clinical pre-/post-test probability was already shown in a similar group of patients of the same study population^13,15^ and is consistent with previous studies, e.g. in a German cohort of 1316 patients with stable chest pain.^35,36^ Furthermore, we could confirm the published results of the diagnostic performance of the Ca-score alone.^37^

Cardiac troponin and the coronary artery Ca-score as stand-alone variables are related to MACE and all-cause death as has been extensively demonstrated.^11,15,33,38–41^ Not known before was that despite the interrelation of these two markers,^18,42^ their combined prognostic value is even higher. A recent study of Razavi *et al*.^43^ demonstrated an additional prognostic value of hs-TnT <3ng/mL in combination with a Ca-score of 0 in patients with diabetes mellitus or metabolic syndrome to maintain this value over a period of 10 years.

Our seventh finding indicates that the Ca-score is only predictive for coronary artery-related vascular events, whereas elevated hs-cTnT provided good prognostic information for stroke/TIA and peripheral arterial events in our study population. However, in some previously performed studies using just stroke/TIA as an outcome measure, the Ca-score showed a prognostic value, as was shown by Hermann *et al*. for 4180 patients without known CAD for a 10-year follow-up period.^44–46^ Hermann *et al*. analysed a study population with a lower mean age and the Ca-score showed in a subgroup analysis no prediction for stroke in patients older than 65 years. Hs-cTnT as a possible predictive marker for non-cardiac vascular events has already been shown in prior studies,^47,48^ whereas the mechanism still remains unclear.

Only a very small number of patients had a Ca-Score of 0 but an elevated troponin in the current study. This finding may point to the fact that patients without calcifications in their coronary arteries have a lower biological age than their chronological age^40^ and thus probably are generally healthier than subjects of the same chronological age but with calcified coronary arteries.

### Limitations

This study is a single-centre study, which may limit the possibility to generalize our findings. However, a large real-world cohort of consecutive patients referred for cardiac imaging was evaluated, which should limit this bias.

Although we have a very stringent method for the adjudication of fCAD and this adjudication was performed by two independent cardiologists, we still may have misclassified a small number of patients.

## Conclusion

The Ca-score alone provided good diagnostic accuracy for the diagnosis of fCAD, which was not improved by utilizing a combined approach with hs-cTnT/I. However, the prognostic performance of a combination of the Ca-score and hs-cTnT for MACE and all-cause mortality was superior compared with each marker alone.

## Supplementary data

Supplementary data are available at *European Heart Journal - Cardiovascular Imaging* online.

## Supplementary Material

jeae093_Supplementary_Data

## Data Availability

The data underlying this article will be shared on reasonable request to the corresponding author.
