# Supplementary material for: Combining anatomical and biochemical markers in the detection and risk stratification of coronary artery disease
Source: Eur Heart J Cardiovasc Imaging. 2024 Apr 9;25(9):1197–205. doi: 10.1093/ehjci/jeae093 (PMC11346366; doi:10.1093/ehjci/jeae093)
Supplement: jeae093_Supplementary_Data [file jeae093_supplementary_data.docx]

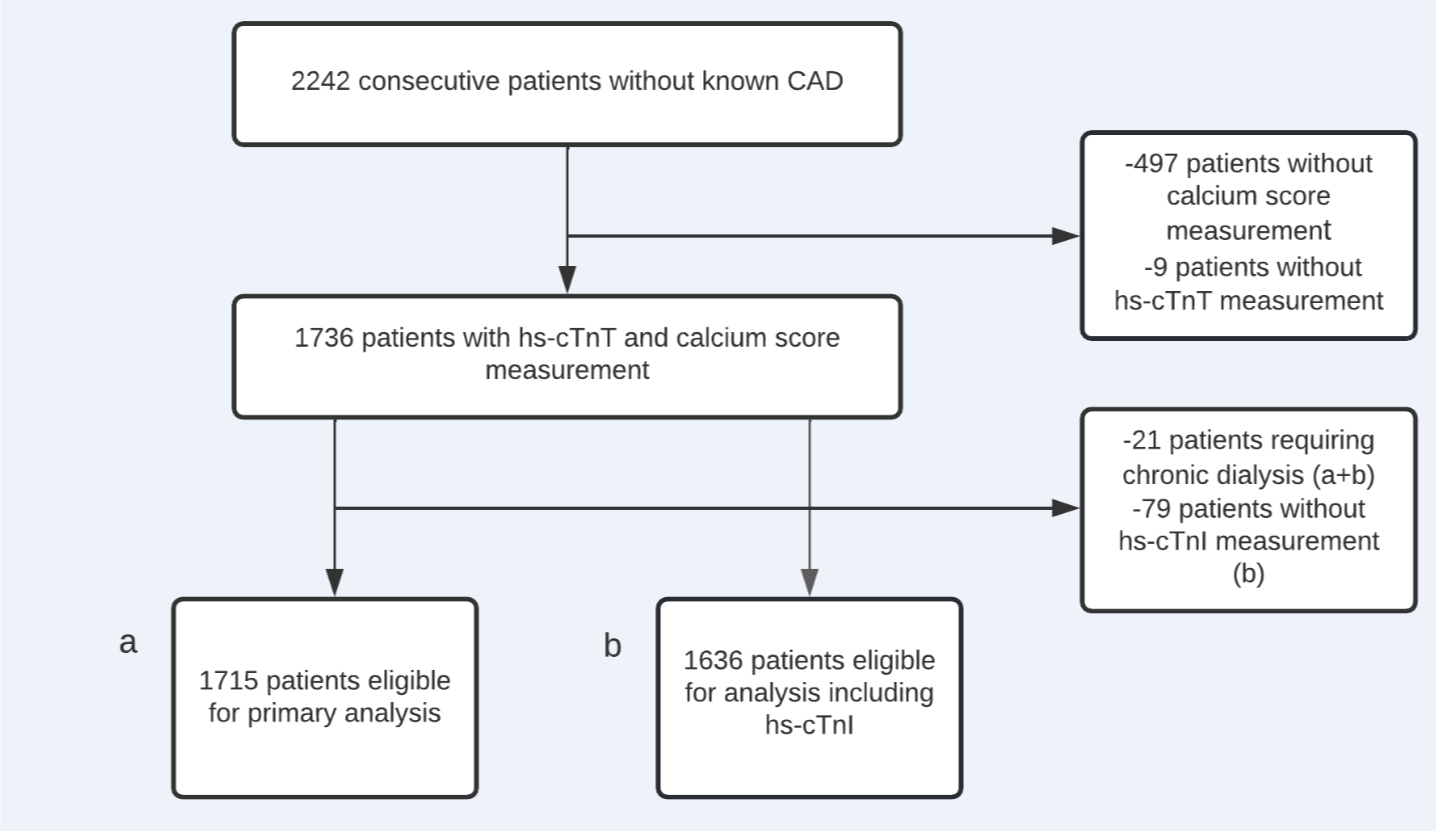


Figure 1 Flowchart study population


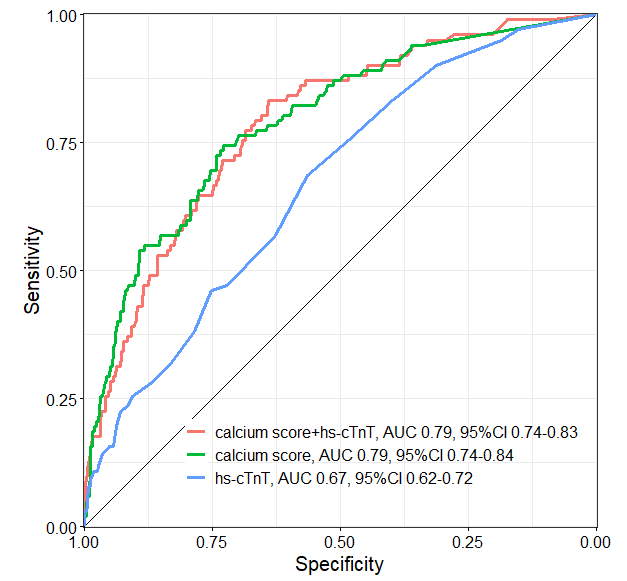


Figure 2 diagnostic accuracy of calcium score, hs-cTnT and a combination of calcium score and hs-TnT, for women; quantified by AUC; n= 753


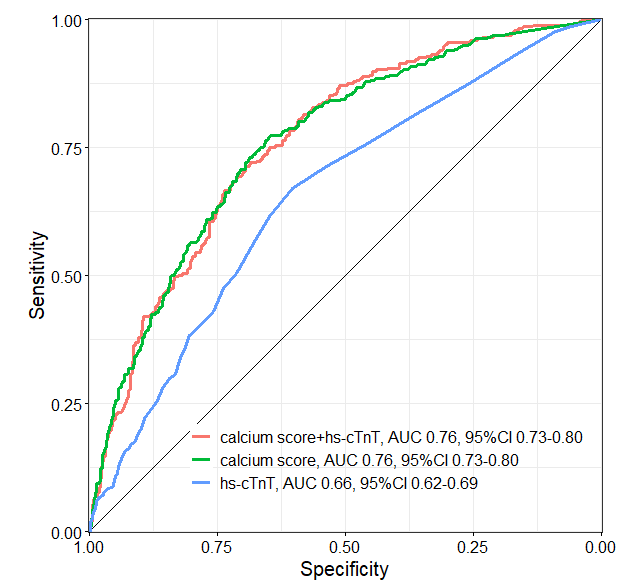


Figure 3 diagnostic accuracy of calcium score, hs-cTnT and a combination of calcium score and hs-cTnT, for men; quantified by AUC; n= 962


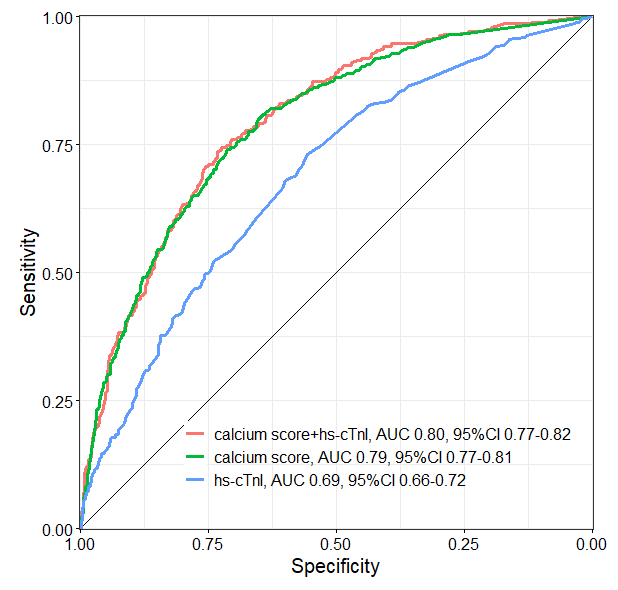


Figure 4 diagnostic accuracy of calcium score, hs-TnI and a combination of calcium score and hs-TnI, quantified by AUC; n= 1636


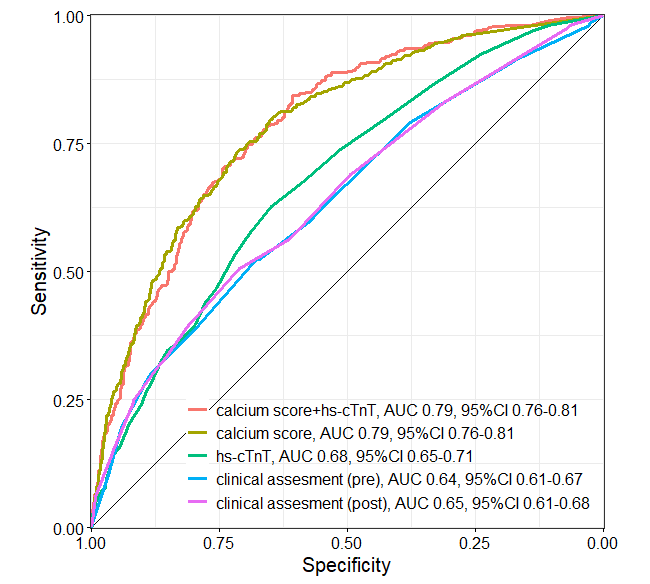


Figure 5 diagnostic accuracy of calcium score, hs-cTnI, VAS clinical assessment before stress/ergometry and VAS clinical assessment after stress/ergometry and a combination of calcium score and hs-cTnI, quantified by AUC; n= 1659

Table 1 Cox- analysis a) without adjustment (n=1709), b) with adjustment (n=1654)* variable is used log2 transformed, Hazard-Ratio (HR) needs to be interpreted accordingly per twofold increase

a)

| Endpoint | Variable | HR (95%CI) | p-value |
| --- | --- | --- | --- |
| 1) AMI and cardiovascular death | Hs-cTnT * | 1.54 (1.29-1.84) | <0.001 |
|  | Calcium score * | 1.11 (1.03-1.19) | 0.005 |
| 2)1) overall death | Hs-cTnT * | 1.68 (1.43-1.96) | <0.001 |
|  | Calcium score * | 1.15 (1.06-1.25) | 0.001 |
| 2)2) AMI, cardiovascular death, late revascularization | Hs-cTnT * | 1.331(1.14-1.55) | <0.001 |
|  | Calcium score * | 1.16 (1.09-1.24) | <0.001 |
| 2)3) Stroke/TIA and peripheral arterial event | Hs-cTnT * | 1.36 (1.13-1.64) | 0.001 |
|  | Calcium score * | 1.09 (1.03-1.164) | 0.006 |

b)

| Endpoint | Parameter | HR (95%CI) | p-value |
| --- | --- | --- | --- |
| 1) AMI and cardiovascular death | Hs-cTnT * | 1.29 (0.99-1.69) | 0.065 |
|  | Calcium score * | 1.08 (1.00-1.17) | 0.044 |
|  | Age | 1.02 (0.99-1.06) | 0.140 |
|  | Sex | 0.74 (0.40-1.35) | 0.320 |
|  | Cystatin C * | 1.86 (0.99-3.50) | 0.054 |
|  | History of cardiovascular disease | 1.03 (0.58-1.82) | 0.921 |
| 2)1) overall death | Hs-cTnT * | 1.33 (1.04-1.70) | 0.024 |
|  | Calcium score * | 1.12 (1.02-1.23) | 0.015 |
|  | Age | 1.05 (1.01-1.08) | 0.007 |
|  | Sex | 0.68 (0.38-1.22) | 0.199 |
|  | Cystatin C * | 2.54 (1.44-4.48) | 0.001 |
|  | History of cardiovascular disease | 0.70 (0.40-1.23) | 0.213 |
| 2)2) AMI, cardiovascular death, late revascularization | Hs-cTnT * | 1.20 (0.97-1.49) | 0.087 |
|  | Calcium score * | 1.15 (1.08-1.24) | <0.001 |
|  | Age | 1.00 (0.98-1.03) | 0.960 |
|  | Sex | 0.78 (0.49-1.25) | 0.307 |
|  | Cystatin C * | 1.75 (1.05-2.92) | 0.032 |
|  | History of cardiovascular disease | 0.98 (0.63-1.53) | 0.919 |
| 2)3) Stroke/TIA and peripheral arterial event | Hs-cTnT * | 1.37 (1.06-1.79) | 0.018 |
|  | Calcium score * | 1.07 (1.00-1.14) | 0.064 |
|  | Age | 1.00 (0.97-1.03) | 0.989 |
|  | Sex | 1.30 (0.75-2.25) | 0.345 |
|  | Cystatin C * | 0.73 (0.37-1.44) | 0.363 |
|  | History of cardiovascular disease | 5.26 (2.97-9.31) | <0.001 |

Table 2 baseline characteristics according to hs-TnT and calcium score (CS) respective below (-) and above (+) the cut-off value (cut of value CS: <100; cut of value hs-cTnT<14 ng/l).

|  | Overall  n=1709 | Hs-cTnT-  Cs-  n=726 | Hs-cTnT-  Cs+  n=507 | Hs-cTnT+  Cs-  n=121 | Hs-cTnT+  Cs+  N=355 |
| --- | --- | --- | --- | --- | --- |
| Sex, male | 957 (56%) | 308 (42.4%) | 335 (66.1%) | 64 (52.9%) | 250 (70.4%) |
| Age | 68 (59-76) | 62 (54-69) | 69 (62.5-75) | 73 (63-79) | 76 (70-80) |
| BMI | 26.9  (24.2-30.5) | 26.8  (24.1-30.7) | 26.8  (24.2-30.4) | 27.4  (24.0-31.3) | 27.0  (24.2-30.4) |
| Symptoms | 1015 (59.4%) | 470 (64.7%) | 303 (59.8%) | 68 (56.2%) | 174 (49%) |
| Pre existing disease |  |  |  |  |  |
| Arterial hypertension | 1216 (71.2%) | 434 (59.8%) | 380 (75%) | 92 (76%) | 310 (87.3%) |
| Hypercholesterolemia | 904 (52.9%) | 335 (46.1%) | 311 (61.3%) | 54 (44.6%) | 204 (57.5%) |
| Arterial Fibrillation | 247 (14.5%) | 64 (8.8%) | 73 (14.4%) | 29 (24%) | 81 (22.8%) |
| Heart failure | 26 (1.5%) | 3 (0.4%) | 4 (0.8%) | 4 (3.3%) | 15 (4.2%) |
| Diabetes | 344 (20.1%) | 114 (15.7%) | 97 (19.1%) | 22 (18.1%) | 111 (31.2%) |
| Peripheral arterial disease | 100 (5.9%) | 13 (1.8%) | 35 (6.9%) | 3 (2.5%) | 49 (13.8%) |
| Status post Stroke/TIA | 129 (7.5%) | 25 (3.4%) | 47 (9.3%) | 11 (9.1%) | 46 (13%) |
| Pacemaker/ICD/CRT | 41 (2.4%) | 11 (1.5%) | 9 (1.8%) | 4 (3.3%) | 17 (4.8%) |
| Family history | 387 (22.6%) | 189 (26%) | 123 (24.3%) | 13 (10.7%) | 62 (17.5%) |
| Malignancy | 254 (14.9%) | 89 (12.3%) | 72 (14.2%) | 17 (14%) | 76 (21.4%) |
| Nicotin use  Stopped use  Active use | 915 (53,5%)  585 (34.2%)  330 (19.3%) | 346 (47,6%)  202 (27.8%)  144 (19.8%) | 307(60,6%)  192 (37.9%)  115 (22.7%) | 54 (44,6%)  41 (33.9%)  13 (10.7%) | 208 (58.6%)  150 (42.3%)  58 (16.3%) |
| Beta blocker | 583 (34.1%) | 203 (28%) | 173 (34.1%) | 42 (34.7%) | 165 (46.5%) |
| ACE-Inhibitor | 359 (21%) | 119 (16.4%) | 98 (19.3%) | 32 (26.4%) | 110 (31%) |
| AT2-antagonists | 526 (30.8%) | 171 (23.6%) | 171 (33.7%) | 45 (37.2%) | 139 (39.2%) |
| Diuretics | 589 (34.5%) | 156 (21.5%) | 181 (35.7%) | 55 (45.5%) | 197 (55.5%) |
| Anticoagulation  Warfarin  New oral  anticoagulants | 233 (13.6%)  156 (9.1%)  77 (4.5%) | 59 (8.2%)  36 (5%)  23 (3.2%) | 66 (14%)  47 (9.3%)  19 (3.7%) | 27 (22.3%)  12 (9.9%)  15 (12.4%) | 81 (22.8%) 61 (17.2%)  20 (5.6%) |
| Aspirin | 1043 (61%) | 222 (30.6%) | 234 (46.2%) | 43 (35.5%) | 167 (47%) |
| Statin | 604 (35.3%) | 186 (25.6%) | 222 (43.8%) | 37 (30.6%) | 159 (44.8%) |
| fCAD | 398 (23.3%) | 48 (6.6%) | 174 (34.3%) | 20 (16.5%) | 156 (43.9%) |
| Hs-cTnT | 8 (5-15) | 5.5 (4-8) | 7 (6-10) | 20 (16-27) | 22 (16-32) |
| Calcium score | 102 (4-478) | 2 (0-27) | 404 (207-824.5) | 23 (1-50) | 627 (290-1366.5) |

Table 3 Cox analysis according to hs-cTnT and calcium score (CS) respective below (-) and above (+) the cut-off value (cut of value CS: <100; cut of value hs-cTnT<14 ng/l). a) without adjustment (n=1709), b) with adjustment (n=1654)*

a)

| Endpoint | Variable | HR (95%CI) | p-value |
| --- | --- | --- | --- |
| 1) AMI and cardiovascular death | Hs-cTnT-, CS+ | 2.73 (1.21-6.12) | 0.015 |
|  | Hs-cTnT+, CS- | 1.34 (0.29-6.22) | 0.705 |
|  | Hs-cTnT+, CS+ | 7.60 (3.62-15.95) | <0.001 |
| 2)1) overall death | Hs-cTnT-, CS+ | 1.76 (0.73-4.24) | 0.210 |
|  | Hs-cTnT-, CS+ | 2.01 (0.54-7.41) | 0.296 |
|  | Hs-cTnT+, CS+ | 9.67 (4.69-19.92) | <0.001 |
| 2)2) AMI, cardiovascular death, late revascularization | Hs-cTnT-, CS+ | 3.78 (2.04-7.00) | <0.001 |
|  | Hs-cTnT+, CS- | 0.86 (0.20-80) | 0.845 |
|  | Hs-cTnT+, CS+ | 7.38 (4.06-13.43) | <0.001 |
| 2)3) Stroke/TIA and peripheral arterial event | Hs-cTnT-, CS+ | 2.05 (0.98-4.30) | 0.057 |
|  | Hs-cTnT+, CS- | 3.56 (1.40-9.04 | 0.008 |
|  | Hs-cTnT+, CS+ | 5.33 (2.72-10.44) | <0.001 |

b)

| Endpoint | Parameter | HR (95%CI) | p-value |
| --- | --- | --- | --- |
| 1) AMI and cardiovascular death | Hs-cTnT-, CS+ | 2.04 (0.85-4.90) | 0.112 |
|  | Hs-cTnT+, CS- | 0.75 (0.15-3.76) | 0.731 |
|  | Hs-cTnT+, CS+ | 3.32 (1.30-8.46) | 0.012 |
|  | Age | 1.03 (1.00-1.06) | 0.064 |
|  | Sex | 0.66 (0.37-1.19) | 0.170 |
|  | Cystatin C * | 2.20 (1.21-3.99) | 0.009 |
|  | History of cardiovascular disease | 1.04 (0.59-1.84) | 0.885 |
| 2)1) overall death | Hs-cTnT-, CS+ | 1.26 (0.50-3.20) | 0.625 |
|  | Hs-cTnT+, CS- | 0.84 (0.21-3.40)) | 0.809 |
|  | Hs-cTnT+, CS+ | 3.07 (1.25-7.52) | 0.014 |
|  | Age | 1.05 (1.02-1.09) | 0.001 |
|  | Sex | 0.57 (0.32-1.00) | 0.051 |
|  | Cystatin C * | 2.94 (1.72-5.02) | <0.001 |
|  | History of cardiovascular disease | 0.73 (0.42-1.27) | 0.265 |
| 2)2) AMI, cardiovascular death, late revascularization | Hs-cTnT-, CS+ | 3.00 ( 1.54-5.87) | 0.001 |
|  | Hs-cTnT+, CS- | 0.59 (0.13-2.72) | 0.501 |
|  | Hs-cTnT+, CS+ | 4.36 (2.07-9.21) | <0.001 |
|  | Age | 1.01 (0.99-1.03) | 0.434 |
|  | Sex | 0.69 (0.43-1.10) | 0.119 |
|  | Cystatin C * | 1.97 (1.21-3.23) | 0.007 |
|  | History of cardiovascular disease | 1.02 (0.66-1.60) | 0.919 |
| 2)3) Stroke/TIA and peripheral arterial event | Hs-cTnT-, CS+ | 1.62 (0.73-3.56) | 0.235 |
|  | Hs-cTnT+, CS- | 2.83 (1.04-7.74) | 0.042 |
|  | Hs-cTnT+, CS+ | 3.41 (1.47-7.88) | 0.004 |
|  | Age | 1.00 (0.97-1.04) | 0.764 |
|  | Sex | 1.12 (0.66-1.91) | 0.675 |
|  | Cystatin C * | 0.79 (0.41-1.50) | 0.468 |
|  | History of cardiovascular disease | 5.38 (3.03-9.54) | <0.001 |


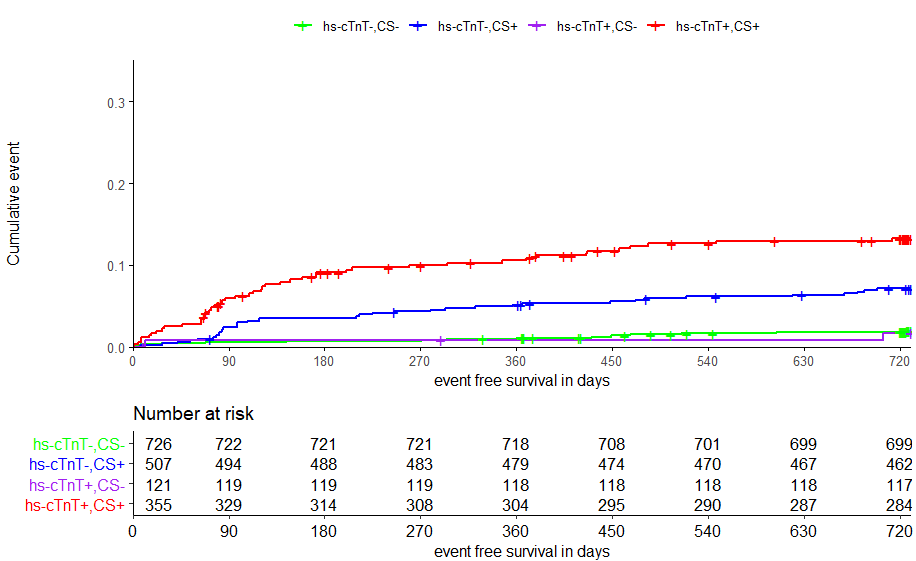


Figure 6 Kaplan-Meier curve for secondary endpoint 2 (cardiovascular death, AMI, late revascularisation). Groups classified by calcium score (CS) and hs-cTnT below (-) and above (+) the cut of value (cut of value CS <100; cut of value hs-cTnT<14 ng/l)


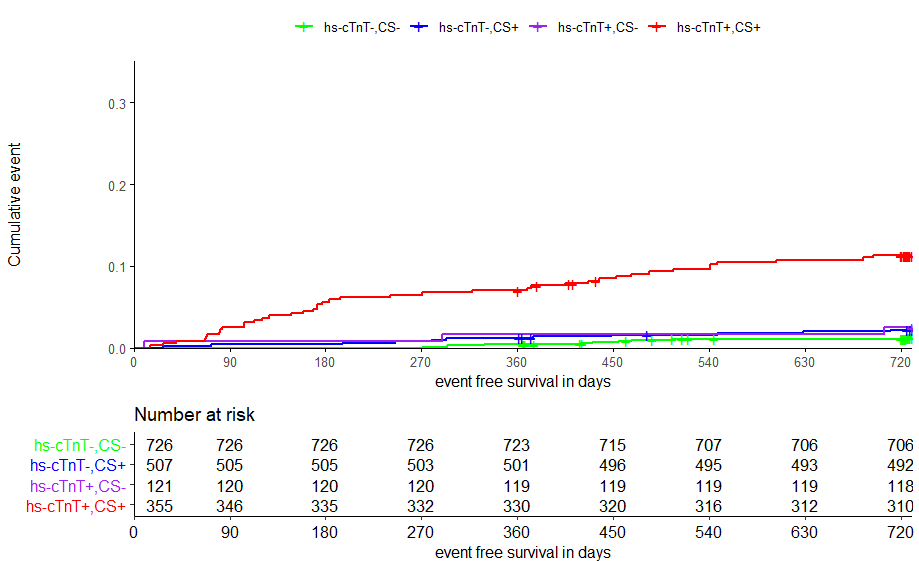


Figure 7 Kaplan-Meier curve for secondary endpoint 3 (stroke/TIA, peripheral arterial event). Groups classified by calcium score (CS) and hs-cTnT below (-) and above (+) the cut of value (cut of value CS <100; cut of value hs-cTnT<14 ng/l)


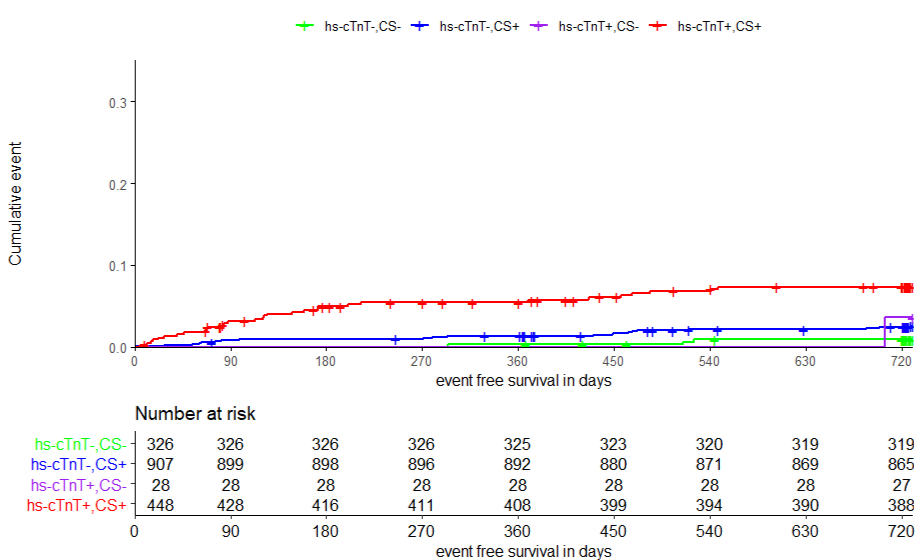


Figure 8 Kaplan-Meier curve for primary endpoint (cardiovascular death, AMI). Groups classified by calcium score (CS) and hs-cTnT below (-) and above (+) the cut of value (cut of value CS=0; cut of value hs-cTnT<14 ng/l)


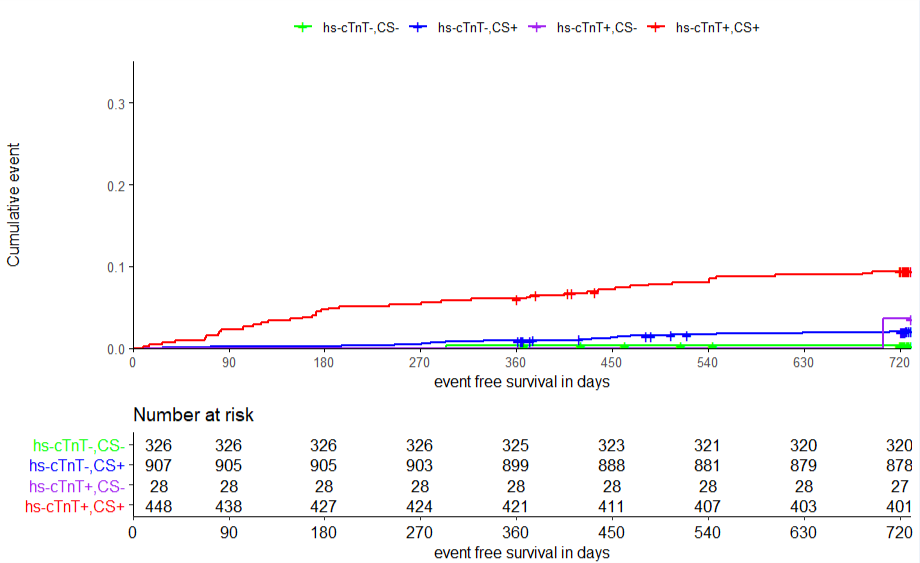


Figure 9 Kaplan-Meier curve for secondary endpoint 1 (all-cause death). Groups classified by calcium score (CS) and hs-cTnT below (-) and above (+) the cut of value (cut of value CS =0; cut of value hs-cTnT<14 ng/l)


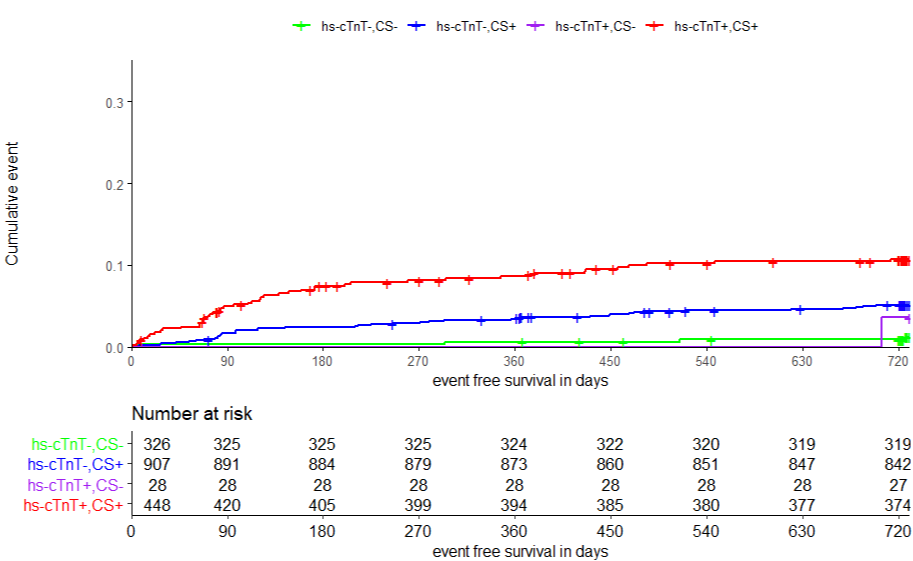


Figure 10 Kaplan-Meier curve for secondary endpoint 2 (cardiovascular death, AMI, late revascularisation). Groups classified by calcium score (CS) and hs-cTnT below (-) and above (+) the cut of value (cut of value CS=0; cut of value hs-cTnT<14 ng/l)


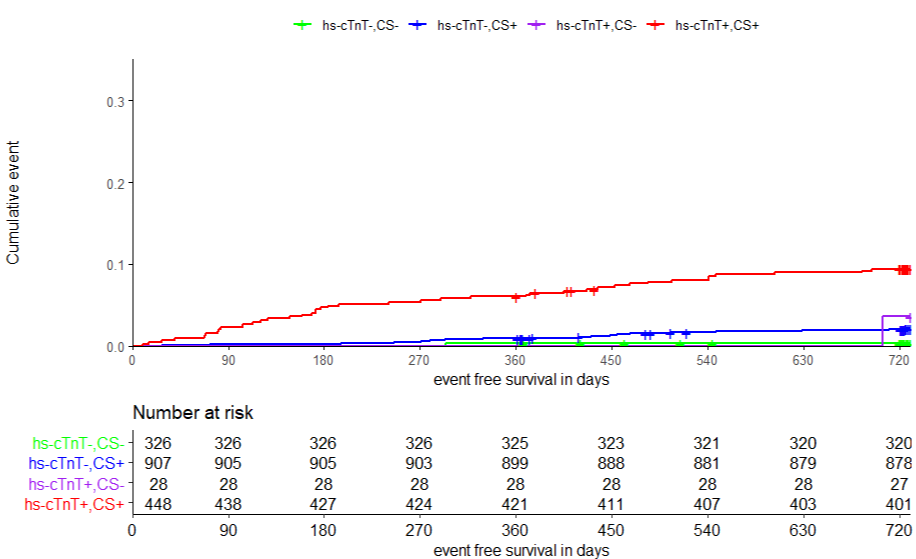


Figure 11 Kaplan-Meier curve for secondary endpoint 3 (stroke/TIA, peripheral arterial event). Groups classified by calcium score (CS) and hs-cTnT below (-) and above (+) the cut of value (cut of value CS =0; cut of value hs-cTnT<14 ng/l)

Appendix methods:

Measurement of Cystatin C

Cystatin C was measured in an external laboratory by SomaLogic Inc Colorado using aptamer-based multiplexed Proteomic Technology, measurement units were relative fluorescent units (RFU). The exact methodology is described elsewhere: In short, it uses a proprietary DNA-based aptamer technology to bind to the target protein with high specificity and the individual protein concentrations are transformed into a corresponding modified aptamer concentration. ^1–3^

1. Gold L, Ayers D, Bertino J, et al. Aptamer-Based Multiplexed Proteomic Technology for Biomarker Discovery. PLoS One [Internet] 2010 [cited 2021 Jan 22];5(12):e15004. Available from: https://dx.plos.org/10.1371/journal.pone.0015004

2. Ganz P, Heidecker B, Hveem K, et al. Development and validation of a protein-based risk score for cardiovascular outcomes among patients with stable coronary heart disease. JAMA - J Am Med Assoc [Internet] 2016 [cited 2021 Feb 9];315(23):2532–41. Available from: https://pubmed.ncbi.nlm.nih.gov/27327800/

3. Zimmermann T, Walter JE, Lopez-Ayala P, et al. Influence of renin-angiotensin-aldosterone system inhibitors on plasma levels of angiotensin-converting enzyme 2. ESC Hear Fail [Internet] 2021 [cited 2022 Feb 26];8(2). Available from: https://pubmed.ncbi.nlm.nih.gov/34596976/
